# Pattern and time point of relapse in locally advanced esophagogastric adenocarcinoma after multimodal treatment: implications for a useful structured follow-up

**DOI:** 10.1007/s00432-023-05254-4

**Published:** 2023-08-17

**Authors:** Ramona Stelmach, Leonidas Apostolidis, Steffen Kahle, Leila Sisic, Henrik Nienhüser, Tim Frederik Weber, Dirk Jäger, Georg Martin Haag

**Affiliations:** 1 Department of Medical Oncology, National Center for Tumor Diseases, Heidelberg University Hospital, Im Neuenheimer Feld 460, 69120 Heidelberg, Germany; 2 Department of Surgery, Heidelberg University Hospital, Heidelberg, Germany; 3 Department of Diagnostic and Interventional Radiology, Heidelberg University Hospital, Heidelberg, Germany; 4 Clinical Cooperation Unit Applied Tumor-Immunity, German Cancer Research Center, Heidelberg, Germany

**Keywords:** Esophagogastric adenocarcinoma, Multimodal treatment, Perioperative chemotherapy, Relapse, Structured follow-up

## Abstract

**Purpose:**

Despite improvements in multimodal treatment of locally advanced esophagogastric adenocarcinoma, the majority of patients still relapses. The impact of structured follow-up for early detection of recurrence is unclear and controversially discussed.

**Methods:**

Patients with locally advanced esophagogastric adenocarcinoma having received neoadjuvant/perioperative chemotherapy followed by tumor resection between 2009 and 2021, underwent a structured follow-up including three-monthly imaging during the first 2 years, followed by semiannual and annual examinations in year 3–4 and 5, respectively. Clinical outcome including pattern and time point of relapse was analyzed.

**Results:**

Two hundred fifty-seven patients were included in this analysis. In 50.2% (*n* = 129) of patients, recurrent disease was diagnosed, with the majority (94.6%) relapsing within the first 2 years. The most common site of relapse were lymph node metastases followed by peritoneal carcinomatosis and hepatic and pulmonary metastases. 52.7% of patients presented with symptoms at the time of relapse. Cumulative risk and time point of relapse differed significantly between patient with a node-positive tumor (ypN+) after neoadjuvant treatment (high-risk group) and patients with node-negative primary tumor (ypN0) (low-risk group). High-risk patients had a significantly inferior disease-free survival (DFS) and overall survival (OS) with 11.1 and 29.0 months, respectively, whereas median DFS and OS were not reached for the low-risk group.

**Conclusions:**

The risk of relapse differs significantly between high- and low-risk patients. Only a part of relapses is associated with clinical symptoms. An individualized follow-up strategy is recommended for high- and low-risk patients considering the individual risk of relapse.

**Supplementary Information:**

The online version contains supplementary material available at 10.1007/s00432-023-05254-4.

## Background

Multimodal treatment has significantly improved outcome in patients with locally advanced esophagogastric adenocarcinoma (EGA) over the last decades. Based on the data of the pivotal phase III MAGIC, CROSS and FLOT-4 trials (Cunningham et al. 2006; van Hagen et al. 2012; Al-Batran et al. 2019), either perioperative chemotherapy or neoadjuvant chemoradiation (for GE junction tumors) followed by primary tumor resection are considered standard of care in Western countries for patients with locally advanced EGA. As the NeoAegis trial demonstrate comparable overall survival (OS) in patients having received perioperative chemotherapy (with ECX being the regimen applied in the majority of patients) or neoadjuvant chemoradiotherapy based on the CROSS protocol, none of these approaches can currently considered to be superior for tumors of the GE junction (Reynolds et al. 2021).

Despite these improvements, the majority of patients relapses and finally dies due to recurrent disease. Most studies report the highest probability of relapse during the first 2 years after the end of primary treatment, whereas relapses after more than 5 years are rarely observed (D'Ugo et al. 2013; Baiocchi et al. 2016; Moorcraft et al. 2016; Lou et al. 2013; Bjerring et al. 2019; Elimova et al. 2017; Kodera et al. 2003). However, the implementation of a standardized and structured follow-up for the early detection of disease recurrence continues to be a matter of controversy, especially since there is no prospective data showing a survival benefit. Recommendations based primarily on retrospective data and expert opinion (Baiocchi et al. 2016). In a retrospective, matched analysis, Sisic et al. could demonstrate an improved OS (84.9 vs. 38.4 months, *p* = 0.040) for intensive follow-up by a specialized center compared to individual follow-up by other physicians (Sisic et al. 2018), whereas a prospective trial by Bjerring et al. did not show a survival benefit for a standardized follow-up including regular imaging (Bjerring et al. 2019).

Recommendations in national guidelines vary from symptom-based assessments to structured follow-up strategies (Ajani et al. 2023; Leitlinienprogramm Onkologie et al. 2022; Lordick et al. 2022; Obermannova et al. 2022). Clear specifications on interval, duration and exact examinations are often missing. In the German and the ESMO (European Society of Medical Oncology) guidelines for esophageal cancer for example, follow-up is primarily intended to detect dysfunction, to assess nutritional status and provide psychological support (Obermannova et al. 2022; Leitlinienprogramm Onkologie et al. 2022). The German and ESMO guidelines on gastric cancer, on the other hand, recommend regular clinical, endoscopic, and imaging examinations also for early detection of recurrences (Leitlinienprogramm Onkologie et al. 2019; Lordick et al. 2022). A report of the European Registration of Cancer Care demonstrated that—although there is no significant evidence of a survival benefit—many European centers use structured follow-up plans including regular cross-sectional imaging and determination of tumor markers for patients with esophageal and gastric cancer after surgery. However, the course of the follow-up varied considerably between the individual countries (Messager et al. 2016).

Among patients having undergone curatively intended multimodal treatment, the risk of relapse varies significantly. Further evaluations of the effect of pathologic tumor response and nodal status on survival in patients treated in the MAGIC trial demonstrated that poor tumor regression and lymph node metastases were negatively related to survival. The presence of lymph node metastases was identified as the only independent predictor of survival after neoadjuvant chemotherapy (Smyth et al. 2016).

In this retrospective analysis, we sought to investigate time point and patterns of relapse to evaluate the role of structured follow-up in patients with locally advanced EGA and to give individualized recommendations on follow-up depending on risk factors.

## Methods

Data from patients undergoing multimodal treatment for locally advanced EGA between 2009 and 2021 were analyzed. All patients had an indication for neoadjuvant or perioperative systemic treatment according to their initial tumor stage (cT ≥ 2 cN0 or any cT category with cN+ cM0) after interdisciplinary discussion. Surgical resection was planned 4–6 weeks after the end of neoadjuvant treatment. The initial tumor stage, localization of the primary tumor, histological subtype, level of the tumor markers CA72-4, CA19-9 and CEA at diagnosis as well as the histopathological TNM stage after neoadjuvant treatment and the histological regression according to the tumor regression grading established by Becker et al. were recorded (Becker et al. 2012).

After surgery, a structured follow-up was recommended, which consisted of two initial computed tomography (CT) scans followed by alternating CT scans and ultrasound examinations quarterly for 2 years and semiannual in the third year. Subsequently, ultrasound examinations were performed semiannually in the fourth and annually in the fifth year of follow-up (supp. Fig. 1). In the event of suspicious symptoms or findings, follow-up was changed at the discretion of the physician. Furthermore ultrasound could be replaced by a CT scan in case of clinically suspected recurrence or poor imaging quality of ultrasound. In some cases, an abdominal magnetic resonance imaging (MRI) was performed instead of a CT scan at the discretion of the physician, for example due to renal insufficiency. Clinical assessments and determination of level of tumor markers were done at each follow-up. In addition, annual esophagogastroduodenoscopy (EGD) was carried out. In cases of R1/RX resection, EGD was recommended every 3 months within the first 2 years after surgical tumor resection. The time point and localization of relapse as well as the diagnostic modalities performed at relapse, the modality leading to the diagnosis of relapse, clinical symptoms and level of tumor markers CA72-4, CA19-9 and CEA at relapse were documented.

According to the results by Smyth et al. (2016), patients with the presence of lymph node metastases after neoadjuvant treatment (ypN+) were categorized as high-risk patients, whereas patients without lymph node involvement (ypN0) were categorized as low-risk patients.

OS was calculated from the day of surgical tumor resection until death from any cause, with censoring of patients known to be alive at the time of last follow-up. Disease-free survival (DFS) was calculated from the day of surgical tumor resection until relapse or death. In patients without recurrence, DFS was censored at the last examination without evidence for a relapse. Both OS and DFS were estimated using the Kaplan–Meier method. Median follow-up was estimated using the reverse Kaplan–Meier method.

The logrank test was used for univariate analysis. The Cox proportional hazard regression model was used for multivariate analysis with backward selection of risk factors. *p* values < 0.05 were defined as significant. Statistical analyses were performed using IBM SPSS statistics version 29.0 and R software package, version 3.6.1. This retrospective study was approved by the local Ethics committee.

## Results

A total of 257 patients were identified with locally advanced EGA who underwent surgical tumor resection and received systemic chemotherapy either as neoadjuvant chemotherapy alone or as perioperative chemotherapy. Detailed patient characteristics are summarized in Table 1. The median age at diagnosis was 61 years (25–85). 73.6% (*n* = 188) of patients were male. The primary tumor was located in the distal esophagus or cardia (type I and II according to Siewert classification) in 57.2% (*n* = 147) of patients, and subcardial (type III according to Siewert classification) or in the stomach in 42.8% (*n* = 110). Histologically, adenocarcinoma of the intestinal type according to Laurén was diagnosed in the majority of patients (63.8%, *n* = 164). Most patients (74.3%, *n* = 191) received perioperative systemic chemotherapy according to the FLOT regimen. There was no significant difference regarding chemotherapy regimens between the two risk groups (suppl. Table 1). A complete histopathological remission after neoadjuvant chemotherapy was detected in 5.1% (*n* = 13) of patients. Lymph node involvement after neoadjuvant chemotherapy (ypN+) was diagnosed in 52.3% (*n* = 137) of the patients. According to Becker’s classification, 30.7% (*n* = 79) of patients were good responders to neoadjuvant chemotherapy (grade 1a or 1b), whereas 63.8% (*n* = 164) had a poor histological response (grade 2 or 3) (Becker et al. 2012).

**Table 1:** Patient characteristics

	Relapsed patients	Non-relapsed patients	All patients
No. of patients	129	128	257
Age at diagnosis (years)			
Median	61	60	61
Range	30–85	25–79	25–85
Sex			
Male	95 (73.6%)	93 (72.7%)	188 (73.2%)
Female	34 (26.4%)	35 (27.3%)	69 (26.8%)
T stage at diagnosis			
cT1/T2	7 (5.4%)	14 (10.9%)	21 (8.2%)
cT3/T4	108 (83.7%)	110 (85.9%)	218 (84.8%)
cTX	14 (10.9%)	4 (3.1%)	18 (7.0%)
N stage at diagnosis			
cN0	12 (9.3%)	18 (14.1%)	30 (11.7%)
cN+	115 (89.1%)	105 (82.0%)	220 (85.6%)
cNX	2 (1.6%)	5 (3.9%)	7 (2.7%)
Primary tumor localization according to Siewert classification			
AEG I/II	71 (55.0%)	76 (59.4%)	147 (57.2%)
AEG III/gastric cancer	58 (45.0%)	52 (40.6%)	110 (42.8%)
Histological subtype			
Intestinal type	77 (59.7%)	87 (68.0%)	164 (63.8%)
Diffuse type	26 (20.2%)	25 (19.5%)	51 (19.8%)
Others	26 (20.2%)	16 (12.5%)	42 (16.3%)
ypT stage			
ypT0	0	16 (12.5%)	16 (6.2%)
ypT1/pT2	14 (10.9%)	49 (38.3%)	63 (24.5%)
ypT3/pT4	115 (89.1%)	63 (49.2%)	178 (69.3%)
ypN stage			
ypN0	31 (24.0%)	89 (69.5%)	120 (46.7%)
ypN1	22 (17.1%)	18 (14.1%)	40 (15.6%)
ypN2	29 (22.5%)	14 (10.9%)	43 (16.7%)
ypN3	47 (36.4%)	7 (5.5%)	54 (21.0%)
Histological response according to Becker’s classification			
Grade 1a	0	14 (10.9%)	14 (5.4%)
Grade 1b	12 (9.3%)	43 (33.6%)	65 (25.3%)
Grade 2	36 (27.9%)	29 (22.7%)	55 (21.4%)
Grade 3	71 (55.0%)	38 (29.7%)	109 (42.4%)
Unknown	10 (7.8%)	4 (3.1%)	14 (5.4%)
Tumor resection			
R0	108 (83.7%)	128 (100%)	236 (91.8%)
R1	16 (12.4%)	0	16 (6.2%)
RX	5 (3.9%)	0	5 (1.9%)
Histopathological complete remission (ypT0 pN0)			
	0	13 (10.2%)	13 (5.1%)
Perioperative treatment regimen			
FLOT^a^	88 (68.2%)	103 (80.5%)	191 (74.3%)
FLO/FOLFOX^b^	20 (15.5%)	15 (11.7%)	35 (13.6%)
ECX/F or EOX/F	17 (13.2%)	9 (7.0%)	26 (10.1%)
Other platinum based regimens^c^	4 (3.1%)	1 (0.8%)	5 (1.9%)

*FLOT* 5-fluorouracil plus leucovorin, oxaliplatin, docetaxel; *FLO/FOLFOX* 5-fluorouracil plus leucovorin, oxaliplatin; *ECX/F* epirubicin, cisplatin, capecitabine (X) or 5-fluorouracil (F); *EOX/F* epirubicin, oxaliplatin, capecitabine (X) or 5-fluorouracil (F)

^a^Including 12 patients with additional investigational agents (PD-1 inhibitor or HER2 directed therapy)

^b^Including 3 patients with additional investigational agents (PD-1 inhibitor or HER2 directed therapy)

^c^Including additional irradiation in 3 patients

Estimated median follow-up of all patients was 46.8 months (95% CI 40.7–52.9%). Relapse of disease was detected in 50.2% of patients (*n* = 129) during the follow-up time. Key relapse parameters are depicted in Table 2. Median age at relapse was 63 years (31–86). In median, relapse occurred 11.9 months after diagnosis (2.9–54.9 months) and 8.7 months (0–52.5 months) after surgical tumor resection.

**Table 2:** Relapse characteristics

	Low-risk group	High-risk group	All patients
	(ypN0)	(ypN+)	
No. of patients	31 (24.0%)	98 (76.0%)	129
Age at relapse			
Median	63	65	63
Range	47–86	31–80	31–86
Symptoms at relapse	16 (51.6%)	52 (53.1%)	68 (52.7%)
Fatigue, weakness	6 (19.4%)	18 (18.4%)	24 (18.6%)
Dysphagia	2 (6.5%)	13 (13.3%)	15 (11.6%)
Weight loss	4 (12.9%)	16 (16.3%)	20 (15.5%)
Pain	6 (19.4%)	15 (15.3%)	21 (16.3%)
Gastrointestinal symptoms	1 (3.2%)	13 (13.3%)	14 (10.9%)
Neurological symptoms	2 (6.5%)	6 (6.1%)	8 (6.2%)
Dyspnea	1 (3.2%)	1 (1.0%)	2 (1.6%)
Others	1 (3.2%)	3 (3.1%)	4 (3.1%)
Localization of relapse			
Local relapse	9 (29.0%)	19 (19.4%)	28 (21.7%)
Lymph nodes	15 (48.4%)	48 (49.0%)	63 (48.8%)
Lung	5 (16.1%)	17 (17.3%)	22 (17.1%)
Liver	5 (16.1%)	22 (22.4%)	27 (20.9%)
Peritoneal	4 (12.9%)	41 (41.8%)	45 (34.9%)
Bones	5 (16.1%)	9 (9.2%)	14 (10.9%)
Brain	2 (6.5%)	5 (5.1%)	7 (5.4%)
Others	8 (25.8%)	18 (18.4%)	26 (20.2%)
Death	15 (48.4%)	74 (75.5%)	89 (69.0%)

**Fig. 1:**
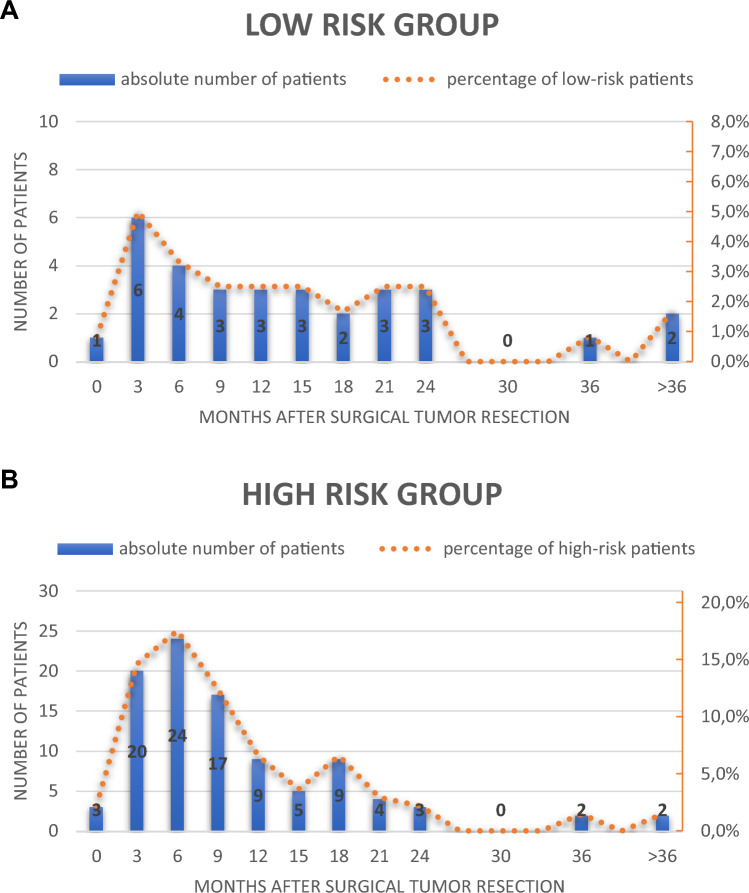
Time of relapse by risk groups. **A** This figure demonstrates the number of relapses in the low-risk group at each follow-up time point in months after surgical tumor resection. The blue bars show the absolute number of relapses using the left y-axis. Absolute numbers of relapses are indicated in each bar. The orange dotted graph demonstrates the relapse rate of each risk group at the corresponding time point using the right y-axis. **B** This figure demonstrates the number of relapses in the high-risk group at each follow-up time point in months after surgical tumor resection. The blue bars show the absolute number of relapses using the left y-axis. Absolute numbers of relapses are indicated in each bar. The orange dotted graph demonstrates the relapse rate of each risk group at the corresponding time point using the right y-axis

As shown in Fig. 1, the majority of patients diagnosed with recurrence (94.6%) relapsed within the first 2 years after surgical tumor resection. The relapse rate of patients at high risk of relapse due to lymph node involvement after neoadjuvant chemotherapy was 71.5% compared to 25.8% in the low-risk group. Within the first 6 months after surgery, 48.0% of all relapses occurred in the high-risk group and 35.5% of all relapses in the low-risk group. After 12 months, 74.5% of all relapses were detected in the high-risk group, compared with 54.8% in the low-risk group.

52.7% of the patients presented tumor-associated symptoms at relapse with pain and weight loss as most common symptoms. The tumor marker CA72-4, CA19-9 and CEA was significantly elevated (≥ 2 upper limit of normal [ULN]) in 34.9%, 28.7%, and 21.7%, respectively. Of the patients with elevated tumor markers at initial diagnosis (≥ 2 ULN), 67.7%, 70.8% and 50.0% of patients also showed elevated tumor markers at recurrence for CA72-4, CA19-9 and CEA, respectively. In 26.3%, 20.0% and 14.9% of cases, tumor markers CA72-4, CA19-9 and CEA were not elevated (< 2 ULN) at initial diagnoses but elevated (≥ 2 ULN) at relapse, respectively.

Until relapse, the median number of cumulative CT scans was 2 (1–8) per patient and the median number of cumulative ultrasounds was 0 (0–8) per patient. In most cases, relapse was diagnosed by a CT scan. From 119 CT scans of the abdomen and 124 CT scans of the chest performed at the time of relapse, 83.2% (*n* = 99) and 52.4% (*n* = 65), respectively, resulted in diagnosis of disease recurrence by detecting tumor manifestations. 15 patients received an ultrasound examination at time of relapse, of which 73.3% (*n* = 11) demonstrated tumor relapse. In a few cases, relapse was confirmed by cerebral magnetic resonance imaging (cMRI), by PET-CT, or by histological confirmation through biopsies in cases of equivocal results based on imaging, e.g., if tumor lesions were suspicious for a second malignant disease or other diagnosis. Additional imaging due to elevated tumor markers was performed in 13.2% of patients (*n* = 17).

In 31 patients, an EGD was performed at the time when a local recurrence was detected in imaging, however, a relapse during endoscopy was only detectable in seven cases.

All patients with R1/RX resection (*n* = 21) developed disease recurrence. Most of these patients (81.0%, *n* = 17) were nodal-positive after neoadjuvant treatment. CT or MRI scans detected relapse in all these patients. Four of these patients showed local recurrence which could be confirmed by EGD in two patients.

The most common tumor localization in relapse was lymph node involvement in 48.8% (*n* = 63), followed by peritoneal involvement in 34.9% (*n* = 45), local recurrence in 21.7% (*n* = 28), hepatic metastases in 20.9% (*n* = 27), pulmonary metastases in 17.1% (*n* = 22), bone metastases in 10.9% (*n* = 14) and cerebral metastases in 5.4% (*n* = 7). Other tumor localizations such as adrenal metastases and soft-tissue metastases were documented in 20.2% of patients (*n* = 26) (Table 2). Most patients (57.7%) with a primary tumor in the distal esophagus or cardia (type I and II according to Siewert classification) presented with lymph node metastases at relapse which were located both above and below the diaphragm followed by liver metastases in 25.4% and other sites in 23.9% such as adrenal glands, soft-tissue and ovary. Patients with a primary tumor located subcardially (type III according to Siewert classification) or in the stomach presented with peritoneal metastases in 58.6% and lymph node metastases in 37.9%, which were mainly located infradiaphragmatically (suppl. Table 2).

**Fig. 2:**
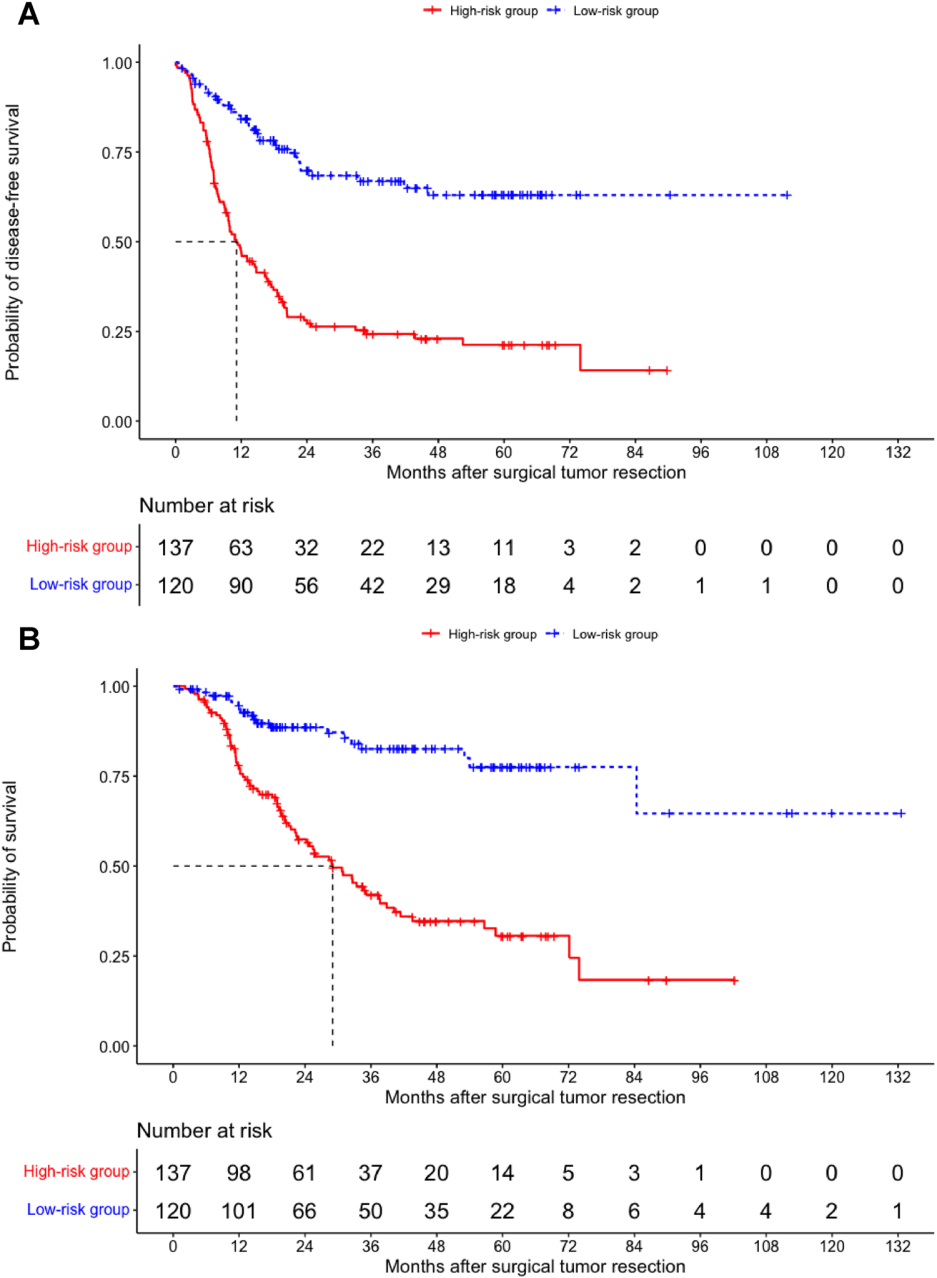
DFS and OS of high-risk patients vs. low-risk patients. **A** Kaplan–Meier survival estimates showing disease-free survival (DFS) after the date of surgical tumor resection for the high-risk (red graph) and the low-risk group (blue graph) including number at risk. The median DFS of the high-risk group is shown with dashed lines. *p* value using the logrank-test was < 0.001. **B** Kaplan–Meier survival estimates showing overall survival (OS) after the date of surgical tumor resection for the high-risk (red graph) and the low-risk group (blue graph). The median OS of the high-risk group is shown with dashed lines. *p* value using the logrank-test was < 0.001

Median DFS and OS of all patients from the date of surgical tumor resection was 20.4 months (95% CI 11.4–29.4 months) and 72.1 months (95% CI 47.2–97.0 months), respectively. Patients with high risk of relapse had a significantly inferior median DFS of 11.1 months (95% CI 8.4–13.8 months) and OS of 29.0 months (95% CI 22.0–36.0), whereas the median DFS and OS of the low-risk group was not reached (each *p* < 0.001) (Fig. 2).

During the follow-up period, 37.7% (*n* = 97) of all patients died. 91.8% (*n* = 89) of them were diagnosed with relapse before.

As shown in Table 3, univariate analysis identified age (< 60y vs. ≥ 60y), ypN status (ypN0 vs. ypN+), histological regression according to Becker’s classification (grade 1a/1b vs. 2/3) and tumor resection status (R0 vs. R1/RX) as significant prognostic factor for DFS. Histological subtype (intestinal vs. others), ypN status (ypN0 vs. ypN+), histological regression according to Becker’s classification (grade 1a/1b vs. 2/3) and tumor resection status (R0 vs. R1/RX) were significantly associated with superior OS. In multivariate analysis, ypN status, tumor resection status and histopathological regression could be identified as independent risk factor for both DFS (ypN+ status: HR 2.72, 95% CI 1.77–4.16, *p* < 0.001; resection status R1/RX: HR 2.82, 95% CI 1.72–4.64, *p* < 0.001; histopathological regression (Becker grade 2/3): HR 3.17, 95% CI 1.74–5.79, *p* < 0.001) and OS (ypN+ status: HR 3.35, 95% CI 1.90–5.88, *p* < 0.001; resection status R1/RX: HR 4.28, 95% CI 2.48–7.37, *p* < 0.001; histopathological regression (Becker grade 2/3): HR 2.75, 95% CI 1.28–5.91, *p* = 0.009).

**Table 3:** Univariate analysis

	*n* (% of patients)	2y-DFS (95% CI)	*p* value	2y-OS (95% CI)	*p* value
Age at diagnosis					
< 60 years	103 (40.1)	56.9 (46.7–67.1)	0.017	75.0 (66.0–84.0)	0.057
≥ 60 years	154 (59.9)	38.6 (30.0–47.2)		68.1 (59.7–76.5)	
Gender					
Male	188 (73.2)	43.9 (36.1–51.7)	0.864	71.2 (63.8–78.6)	0.894
Female	69 (26.8)	52.4 (39.8–65.0)		70.6 (59.2–82.0)	
cT-stage					
T1/2	21 (8.2)	60.3 (35.7–84.9)	0.058	95.0 (85.2–100.0)	0.053
T3/4	218 (84.8)	46.4 (38.0–53.6)		68.7–61.9–75.5)	
cN-stage					
N0	30 (11.7)	54.6 (34.8–74.4)	0.186	82.8 (66.8–98.8)	0.325
N+	220 (85.6)	44.8 (37.8–52.0)		69.5 (62.9–76.1)	
Localization of primary tumor					
EGA I/II	147 (57.2)	47.5 (38.7–56.3)	0.544	73.9 (65.9–81.9)	0.228
EGA III + gastric cancer	110 (42.8)	44.7 (34.7–54.7)		67.7 (58.1–77.3)	
Histological subtype					
Intestinal	164 (63.8)	48.8 (40.4–57.2)	0.272	75.5 (68.1–82.9)	0.010
Others	93 (36.2)	42.0 (31.2–52.8)		64.0 (53.6–74.4)	
ypN-stage					
ypN0	120 (46.7)	69.8 (60.6–79.0)	< 0.001	88.6 (82.4–84.8)	< 0.001
ypN +	137 (53.3)	27.3 (19.3–35.3)		57.5 (48.5–66.5)	
Histological regression					
Grade 1a/1b	69 (26.8)	82.7 (73.1–92.3)	< 0.001	94.9 (89.1–100.0)	< 0.001
Grade 2/3	174 (67.7)	32.7 (24.9–40.5)		62.1 (54.1–70.1)	
Tumor resection status					
R0	236 (91.8)	50.8 (43.8–57.8)	< 0.001	75.5 (69.3–81.7)	< 0.001
R1/RX	21 (8.2)	0		30.3 (9.7–50.9)	

## Discussion

Despite advances in multimodal treatment of patients with locally advanced EGA, more than half of the patients are diagnosed with recurrence during follow-up. However, structured follow-up remains controversial, since prospective data demonstrating an OS benefit are missing. Therefore, recommendations regarding follow-up in national guidelines are not consistent and do often not give specifications on interval, duration and exact examinations.

In multivariate analysis, tumor resection status and histopathological regression were identified as independent prognostic factors in addition to ypN status. Due to the high overlap between R1 resection and the ypN+ status, and taken into consideration that technical aspects in the analysis of the histological regression might significantly differ between different centers (e.g. use of immunohistochemistry to detect residual vital tumor cells) limiting comparability, high-risk patients were defined by ypN status alone. Additionally, the ypN status is already established as a strong prognostic marker (Smyth et al. 2016) and the use of ypN status is more feasible compared to a complex score with multiple parameters.

Consistent with published data, our results demonstrate that most relapses (94.6%) occur within the first 2 years after surgical tumor resection (Baiocchi et al. 2016; Elimova et al. 2017; Bjerring et al. 2019; Lou et al. 2013; Moorcraft et al. 2016; D'Ugo et al. 2013; Kodera et al. 2003). In the high-risk group, the recurrence rate was significantly higher (71.5%) compared to the low-risk group (25.8%) and relapses tend to occur earlier in the high-risk group. Therefore, a structured follow-up with the intention of an early detection of potential disease recurrence seems advisable in the first 2 years after surgical tumor resection, especially for the high-risk group.

Considering the examination modalities that led to the diagnosis of disease recurrence, our data suggest that cross-sectional imaging, which mean CT or MRI, has the highest sensitivity for detecting recurrence and lead to diagnosis of recurrence in most patients. The benefit of ultrasound examinations seems limited and was only beneficial for a small proportion of patients. Since there was no patient in whom tumor manifestations could be seen only in the EGD, regular EGD has limited value for the detection of potential recurrences and should rather be performed in cases of suspected dysfunction or to clarify postoperative complaints. Furthermore, even in patients having undergone RX or R1 resection, EGD did not show superiority over cross-sectional imaging in terms of detection of a local recurrence.

Regular PET-CT scans were not performed since statutory health insurance do not cover PET-CT scans for follow-up of EGA patients in Germany. Furthermore, sensitivity of PET-CT especially for patients with peritoneal metastasis or signet ring cell adenocarcinoma is not significantly superior compared to contrast CT scan (Gertsen et al. 2021; Filik et al. 2015; Sim et al. 2009; De Potter et al. 2002). In addition, recent phase III trials regarding multimodal treatment in locally advanced EGA such as the FLOT 4 trial or the MAGIC trial did not use regular PET-CT scans for follow-up. Thus, PET-CT scans in our cohort were only performed in cases of clinical or laboratory suspicion of recurrence when CT findings were inconclusive.

Localization of metastatic sites in dependence to the location of the primary tumor showed a trend toward increased infradiaphragmatic lymph node metastases and peritoneal metastases for subcardial primary tumors. However, because this trend is not significant and more than a quarter of patients also showed supradiaphragmatic metastases, thoracic imaging should not be omitted. Also, in type I and II tumors according to the Siewert classification, metastases were localized both, supra- and infradiaphragmatically, so cross-sectional imaging of the chest and abdomen should be performed as a standard procedure.

Since the level of tumor markers in the group of patients without recurrence during follow-up and their consequences were not systematically investigated in this study, no definite statement can be made based on our results and previously published data on the significance of tumor markers in the follow-up of EGA. In about 10% of the relapsed patients, additional imaging due to increasing tumor markers resulted in the diagnosis of a disease recurrence. In addition, the majority of patients with initially elevated tumor markers also showed elevated tumor markers at the time of relapse. These aspects could indicate that the determination of tumor markers in the follow-up might be beneficial for a subgroup of patients. However, more detailed analyses on larger patient cohorts regarding the significance of tumor markers are needed.

To date, there is no evidence of a survival benefit for structured follow-up resulting from prospective studies. In the prospective EUFURO study by Bjerring et al., the comparison of standard clinical assessments without regular imaging to regular EGD and PET-CT for patients with upper gastrointestinal cancer after surgery could not demonstrate a prolonged overall survival for regular imaging. However, it must be considered that this study also included patients with pancreatic cancer, who certainly have a worse prognosis compared to EGA patients, and since the data collection period was between 2011 and 2014, patients only received chemotherapy without targeted agents as second-line systemic treatment. Most patients with relapse in this study did not receive any cancer treatment but were treated according to best supportive care concepts (Bjerring et al. 2019).

In a retrospective analysis of 119 patients with recurrence of EGA after multimodality therapy, Apostolidis et al. showed that the prognosis of patients is very heterogeneous. Prognosis was significantly improved when local treatment options were available, which could be performed in 23% of patients (Apostolidis et al. 2023). These data indicate that structured follow-up is needed to identify patients with mostly asymptomatic, unilocular relapse who are suitable for local therapies. Sisic et al. could demonstrate a survival benefit for those patients eligible for surgical resection of recurrence, especially when combined with chemo- and/or radiotherapy (Sisic et al. 2018).

Systemic salvage treatment options currently include cytotoxic chemotherapy, as well as targeted agents and checkpoint inhibitors depending on the treatment-free interval as well as tissue-based biomarkers. (Thuss-Patience et al. 2011; Guimbaud et al. 2014; Hironaka et al. 2013; Wainberg et al. 2021; Sun et al. 2021; Janjigian et al. 2021; Bang et al. 2010; Shitara et al. 2020; Wilke et al. 2014). The emerging field of new treatment options might result in an increased benefit if the relapse is diagnosed as long as the patient is capable to receive a tumor-specific therapy.

So far, the German S3 and ESMO guideline on esophageal cancer recommends symptom-based follow-up primarily with regards to detect functional disorders, to assess the nutritional status, and for psychological support, as data regarding an OS benefit for early detection of recurrence are lacking (Obermannova et al. 2022; Leitlinienprogramm Onkologie et al. 2022). Recommendations for gastric cancer include the intention of early detection of recurrence. The German S3 guideline recommends clinical, endoscopic and imaging controls at least semiannually for 2 years followed by at least annual intervals up to 5 years after surgery (Leitlinienprogramm Onkologie et al. 2019). The ESMO guideline on gastric cancer recommends individualized follow-up care tailored to the patient and the stage of disease, without providing specific guidelines on how to proceed (Lordick et al. 2022).

Overall, structured follow-up for early detection of potential disease recurrence appears to be reasonable in terms of prognosis and quality of life, as patients with early recurrence detection often have better general health conditions, reduced disease-related symptom burden, and, thus, better treatment tolerance. In addition, local interventions can be applied more frequently and might result in disease control without the necessity of systemic therapies, thereby avoiding treatment-related side effects. However, a lead-time-bias (earlier diagnosis of a relapse without a benefit in terms of absolute survival) cannot be excluded. Thus, randomized studies are needed to define the role of imaging-based follow-up.

**Fig. 3:**
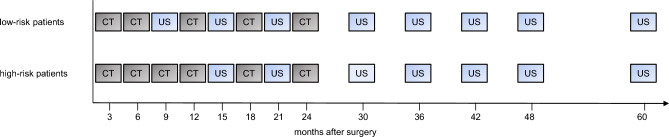
Proposed risk-adapted imaging for structured follow-up. For patients at low risk of recurrence, after two initial CT scans, alternating ultrasound examinations and CT scans are recommended at 3-monthly intervals for up to 2 years after surgery. For patients at high risk of recurrence, 3 monthly CT scans for 1 year followed by alternating ultrasound examinations and CT scans in the second year after surgery are recommended. Beyond 2 years after surgery, regular ultrasound examinations are recommended, initially at 6 monthly intervals, then annually after 4 years, for both risk groups. Cross-sectional imaging should be performed in case of suspicious symptoms or abnormalities in ultrasound examinations

Based on the data presented, we propose a structured, risk-adapted follow-up with regular cross-sectional imaging for the first 2 years after surgery (Fig. 3). Patients at high risk of relapse should undergo cross-sectional imaging with a CT scan every 3 months in the first year after surgery, followed by alternating ultrasound examinations and CT scans in the second year after surgery. In patients at low risk of relapse, two initial CT scans at 3-montly intervals are recommended followed by alternating ultrasound examinations and CT scans every 3 months for up to 2 years after surgery.

Since our data do not support regular cross-sectional imaging beyond 2 years after surgery, a subsequent, symptom-based follow-up with ultrasound examinations might be reasonable for up to 5 years after surgery. Cross-sectional imaging should be performed in case of suspicious symptoms or abnormalities in ultrasound examinations. Due to the limited value of endoscopy in detecting relapse, EGDs should only be performed when clinically indicated.

Limitations of our analysis include that only patients who received surgical tumor resection were included in our analysis. In addition, only the follow-up of patients with recurrences was systematically analyzed. Furthermore, multicenter or nationwide data would be desirable, but multicenter registries including follow-up data do not exist in Germany. However, our outcome data show comparable results to other retrospective analyses from German centers after multimodal treatment, so that our data can be considered representative (Favi et al. 2017; Gebauer et al. 2023; Glatz et al. 2020).

In summary, the results of our evaluation argue for risk-adapted structured follow-up, especially in the first 2 years after surgical tumor resection, for early detection of disease recurrence to enable optimized treatment for these patients. However, prospective, multicenter studies proving a benefit of structured follow-up in terms of survival and quality of life are still lacking.

### Supplementary Information

Below is the link to the electronic supplementary material.

Supplementary file1 (PDF 45 KB)

Supplementary file2 (DOCX 14 KB)

Supplementary file3 (DOCX 14 KB)

## Data Availability

The datasets analyzed during the current study are available from the corresponding author on reasonable request.
