# Supplementary figures and images for: Pattern and time point of relapse in locally advanced esophagogastric adenocarcinoma after multimodal treatment: implications for a useful structured follow-up
Source: J Cancer Res Clin Oncol. 2023 Aug 17;149(16):14785–96. doi: 10.1007/s00432-023-05254-4 (PMC10602954; doi:10.1007/s00432-023-05254-4)

**Supplementary Figure 1: Scheme of structured follow-up**

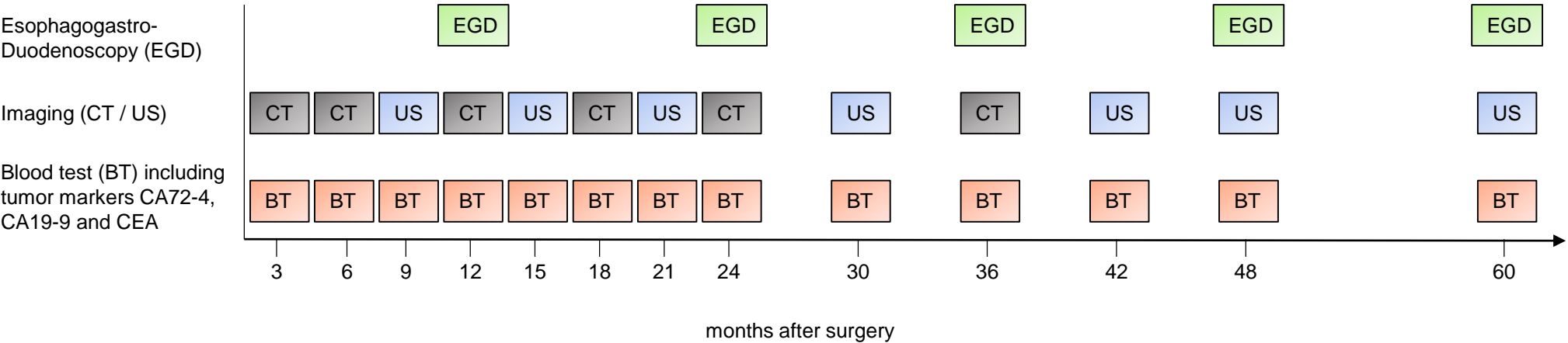

Supplement: Supplementary file 1 — Supplementary file1 (PDF 45 KB) [file 432_2023_5254_MOESM1_ESM.pdf]
