# Supplementary material for: Pattern and time point of relapse in locally advanced esophagogastric adenocarcinoma after multimodal treatment: implications for a useful structured follow-up
Source: J Cancer Res Clin Oncol. 2023 Aug 17;149(16):14785–96. doi: 10.1007/s00432-023-05254-4 (PMC10602954; doi:10.1007/s00432-023-05254-4)
Supplement: Supplementary file 2 — Supplementary file2 (DOCX 14 KB) [file 432_2023_5254_MOESM2_ESM.docx]

|  | Low-risk group | High-risk group | All patients |
| --- | --- | --- | --- |
| No. of patients | 120 | 137 | 257 |
| Perioperative treatment regimen  FLOT  FLO/FOLFOX  ECX/F or EOX/F  other platinum based regimens | 94 (78.3%)  12 (10.0%)  12 (10.0%)  2 (1.7%) | 97 (70.8%)  23 (16.8%)  14 (10.2%)  3 (2.2%) | 191 (74.3%)  35 (13.6%)  26 (10.1%)  5 (1.9%) |

**Supp. Table 1: Perioperative chemotherapy-regimens by risk groups**
