# Supplementary material for: Pattern and time point of relapse in locally advanced esophagogastric adenocarcinoma after multimodal treatment: implications for a useful structured follow-up
Source: J Cancer Res Clin Oncol. 2023 Aug 17;149(16):14785–96. doi: 10.1007/s00432-023-05254-4 (PMC10602954; doi:10.1007/s00432-023-05254-4)
Supplement: Supplementary file 3 — Supplementary file3 (DOCX 14 KB) [file 432_2023_5254_MOESM3_ESM.docx]

**Supp. Table 2: Sites of recurrence in relation to the location of the primary tumor**

|  | AEG I/II | AEG III or gastric cancer |
| --- | --- | --- |
| No. of patients | 71 | 58 |
| Site of recurrence  local relapse  liver  lymph nodes  supradiaphragmatic  infradiaphragmatic  lung  bone  peritoneum  brain  other | 16 (22.5%)  18 (25.4%)  41 (57.7%)  23 (32.4%)  23 (32.4%)  13 (18.3%)  10 (14.1%)  11 (15.5%)  5 (7.0%)  17 (23.9%) | 12 (20.7%)  9 (15.5%)  22 (37.9%)  5 (8.6%)  20 (34.5%)  9 (15.5%)  4 (6.9%)  34 (58.6%)  2 (3.4%)  9 (15.5%) |
